# Supplementary material for: Multifunctional Freestanding Microprobes for Potential Biological Applications
Source: Sensors (Basel). 2019 May 20;19(10):2328. doi: 10.3390/s19102328 (PMC6567016; doi:10.3390/s19102328)
Supplement: Supplementary file 1 [file sensors-19-02328-s001.zip › supplementary/Supplementary.doc]

**Supplementary Materials**

***Sensors***

**Multifunctional three-dimensional microprobes for potential biological applications**

Nana Yang^a^, Zhenhai Wang^a^, Jingjing Xu^a,b^, Lijiang Gui^c^, Zhiqiang Tang^a^, Yuqi Zhang^d^, Ming Yi^d^, Shuanglin Yue^a,*^, and Shengyong Xu^a,*^

^a^ Key Laboratory for the Physics & Chemistry of Nanodevices, and Department of Electronics, Peking University, Beijing 100871, China.

^b^ Institute of Microelectronics, Shandong University, Jinan 250100, P. R. China.

^c^ Department of Micro-Nano Fabrication Technology, Institute of Electrical Engineering, Chinese Academy of Sciences, Beijing 100190, China.

^d^ Neuroscience Research Institute, and Department of Neurobiology, School of Basic Medical Sciences, Peking University, Beijing 100083, P. R. China.

*Authors to whom correspondence should be addressed. E-mails: [slyue@pku.edu.cn](mailto:slyue@pku.edu.cn) (SLY) [xusy@pku.edu.cn](mailto:xusy@pku.edu.cn) (SYX), Tel: +86-10-62745072, Fax: +86-10-62757555

Experiments on live animals were performed. Adult male Sprague-Dawley rats (300–350 g) were provided by the Department of Laboratory Animal Sciences, Peking University Health Science Center (Beijing, China). Rats were maintained under a constant 12/12-h light/dark cycle with free access to food and water. All experimental procedures were approved by the Animal Care and Use Committee of Peking University, according to the guidelines of the International Association for the Study of Pain. In the surgery processes, the rat was initially anesthetized with 1% sodium pentobarbital (0.10 g/kg, i.p.) and anesthesia was maintained using isoflurane (around 2%) in pure O_2_ inhalation. The head of the rat was fixed in a stereotaxic frame (RWD, Shenzhen, China) using ear-bars. For the brain insertion (Video S1), the probe was inserted in the ventral posterolateral nucleus of the thalamus [anterior/posterior (AP) -2.5 mm, mediolateral (ML) -3.2 mm, dorsoventral (DV) -5.0 mm from bregma]. A small craniotomy was performed to allow the insertion of the probe that was slowly lowered vertically to the depth. For the abdomen insertion, the probe was inserted along the groin of the rat with a depth of 10 mm. For the muscle insertion, the probe was inserted into the thigh of the rat with a depth of 15 mm.

Figure S1	Testing of the freestanding 3D microprobes on live rats, where the probe swards were inserted into tissues of (a) brain, (b) abdomen and (c) thigh muscles, to deep levels of 5-20 mm.
